# Renal cell carcinoma metastasis to the maxillary bone successfully treated with surgery after vascular embolization: a case report

**DOI:** 10.1186/s13256-020-02522-6

**Published:** 2020-10-12

**Authors:** Naoto Nishii, Hiroaki Shimamoto, Toshimitsu Ohsako, Misaki Yokokawa, Yuriko Sato, Yae Ohata, Kou Kayamori, Tohru Ikeda, Hiroyuki Harada

**Affiliations:** 1 Department of Oral and Maxillofacial Surgery, Graduate School of Medical and Dental Sciences, Tokyo Medical and Dental University, 1-5-45 Yushima, Bunkyo-ku, Tokyo, 113-8549 Japan; 2 Department of Oral Pathology, Graduate School of Medical and Dental Sciences, Tokyo Medical and Dental University, 1-5-45 Yushima, Bunkyo-ku, Tokyo, 113-8549 Japan

**Keywords:** Renal cell carcinoma, Metastasis, Oral cavity, Maxillary bone, Pulsatile lesion, Preoperative vascular embolization

## Abstract

**Background:**

Metastasis of renal cell carcinoma to the oral cavity is rare. Renal cell carcinoma metastases are regarded as radioresistant tumors and surgery is recommended. However, since metastatic renal cell carcinoma has poor prognosis and is composed of abundant blood vessels, it is sometimes difficult for clinicians to choose surgical therapy. Here, we report a case of a patient with renal cell carcinoma metastasis to the maxillary bone, which was successfully controlled by surgical therapy after vascular embolization, and provide a detailed literature review regarding the treatments and outcomes of renal cell carcinoma metastasis to the oral cavity.

**Case presentation:**

An 89-year-old Japanese man presented with an 8 × 8-mm granulomatous tumor with palpable pulsation in the left upper gingiva, which had been clinically suspected as an arteriovenous malformation or neoplastic lesion with rich blood vessels. Our patient had undergone left nephrectomy for clear cell carcinoma 7 years prior. Pulmonary metastasis had appeared 3 years later. After intravascular embolization, our patient underwent tumor resection of the maxilla with little intraoperative blood loss. The tumor was diagnosed on histopathology as a metastasis of clear cell renal cell carcinoma to the maxillary bone. Seventeen months after surgery, he died because of pulmonary metastasis without evidence of recurrence in the oral cavity.

**Conclusions:**

Our literature review reveals that oral metastatic lesions of renal cancer often exhibit rapid enlargement and cause severe symptoms, such as dysphagia and bleeding. Although oral metastasis of renal cell carcinoma has a poor prognosis due to the presence of concurrent disseminated metastases, surgical therapy may be recommended because of its high local control rate and ability to maintain quality of life. Preoperative vascular embolization is considered to be effective to reduce intraoperative hemorrhage, which leads to safe surgery.

## Background

Tumor metastasis to the oral cavity is uncommon, comprising only 1% of all oral malignant tumors [1]. After lung and breast carcinoma, renal carcinoma is the third most common tumor that metastasizes to the head and neck [2]. Because oral metastasis of renal cell carcinoma (RCC) is a late-stage phenomenon, often accompanied by lung metastasis, its prognosis is very poor [3]. RCC metastases are often regarded as radioresistant tumors, and surgical treatment is recommended. However, given the poor prognosis of metastatic RCC to the oral cavity, careful consideration is necessary regarding whether surgery can improve quality of life for end-stage oncological patients. In this report, we describe a patient with RCC metastasis that developed in the maxillary bone, which was successfully controlled by surgical treatment after vascular embolization. We provide a review of the current literature and discuss the treatment regarding oral cavity involvement of RCC metastasis.

## Case presentation

**Fig. 1:**
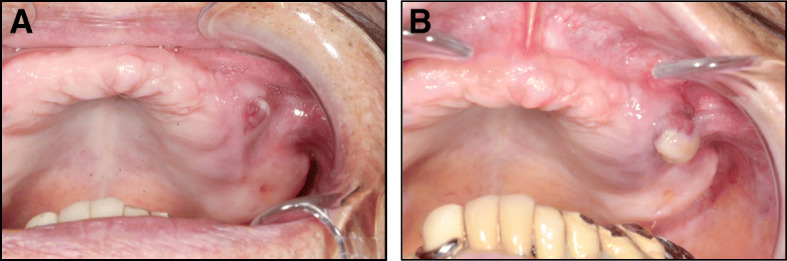
**a** Intraoral photograph of tumor of the left upper gingiva at the first visit, showing granulomatous appearance. **b** Tumor enlarged rapidly in 2 weeks

An 89-year-old Japanese man noticed swelling of the left maxillary gingiva in November 2016. In December 2016, he was referred to our department because the mass had slowly enlarged. His past medical history included clear cell RCC in his left kidney 7 years prior, which had been treated by nephrectomy. Multiple pulmonary metastases of RCC had appeared 3 years after surgery. He had received molecular targeted therapy with sorafenib for 4 years, which suppressed the growth of pulmonary metastases. Intra-oral examination showed an 8 × 8 mm granulomatous tumor with palpable pulsation in the buccal side of the left upper gingiva (Fig. 1a). The lesion enlarged rapidly over 2 weeks (Fig. 1b), and our patient began to feel pain while eating.

**Fig. 2:**
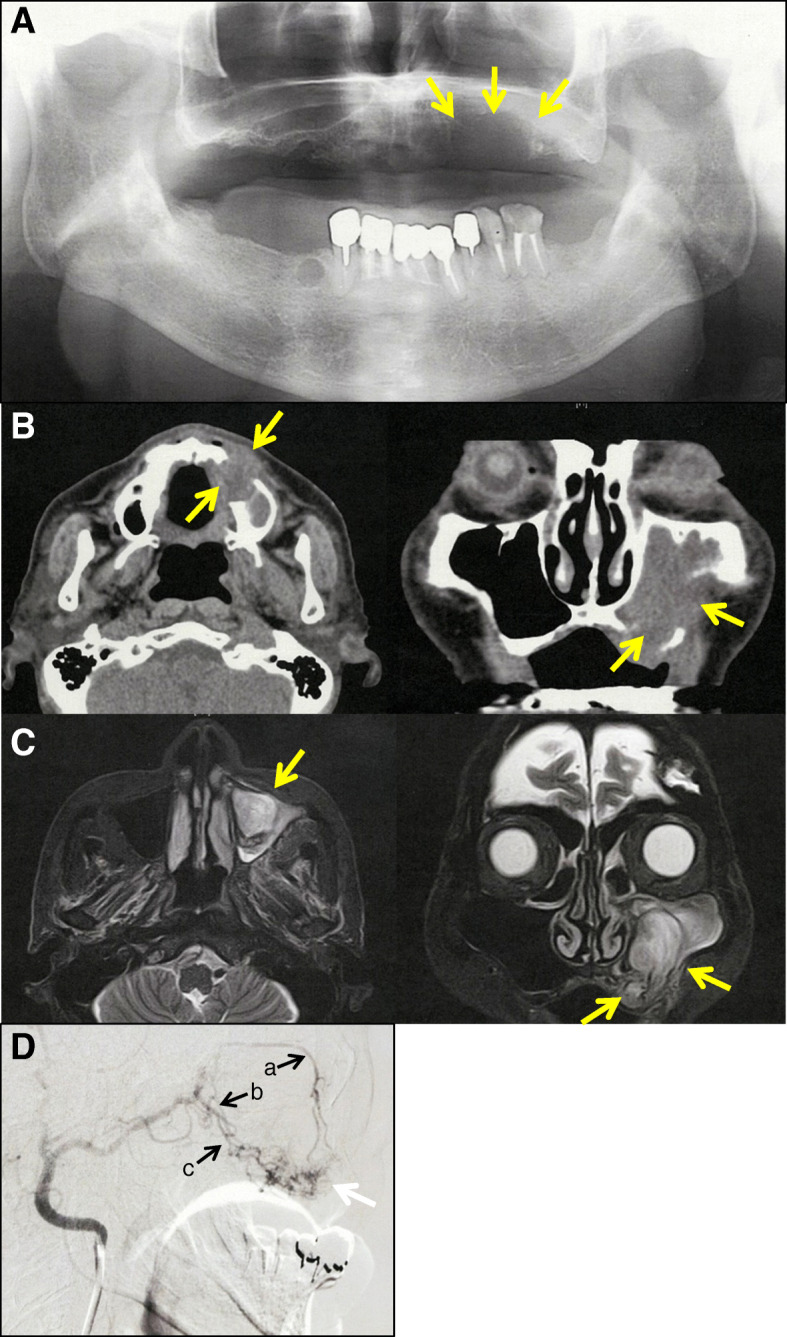
**a** Panoramic radiography revealed resorption of left maxillary alveolar bone (arrow). **b** Enhanced computed tomography showed a tumor destroying the left maxillary bone (arrow). **c** Magnetic resonance imaging showed a 47 × 31 × 22 mm mass in the left maxillary bone and maxillary sinus (arrow). **d** Computed tomography angiography showed a mass with strong enhancement (white arrow) fed by the infraorbital artery (**a**), sphenopalatine artery (**b**), and posterior superior alveolar artery (**c**)

Panoramic radiography revealed resorption of the left maxillary alveolar bone (Fig. 2a). Enhanced computed tomography showed a tumor destroying the left maxillary bone as well as the anterior and lateral walls of the maxillary sinus (Fig. 2b). Magnetic resonance imaging showed a 47 × 31 × 22 mm mass in the left maxillary bone and maxillary sinus, which extended into the oral cavity (Fig. 2c). Computed tomography angiography demonstrated that the mass in the left maxillary bone had strong enhancement and was fed by the infraorbital artery, posterior superior alveolar artery, and sphenopalatine artery (Fig. 2d). Our patient was clinically suspected to have an arteriovenous malformation or neoplastic lesion in the left maxilla.

**Fig. 3:**
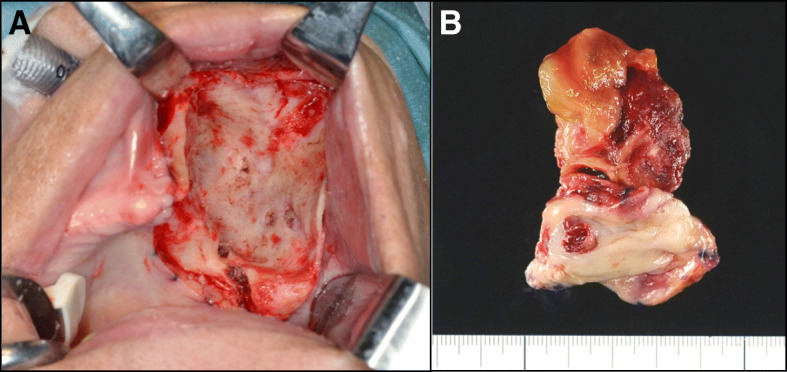
**a** Intraoperative photograph after tumor and surrounding bone were removed. **b** Surgical specimen

In January 2017, 5 days after intravascular embolization of three feeding arteries, our patient underwent maxillary tumor resection. Pulsation around the tumor was not palpable after embolization. Prior to surgery, a biopsy specimen of the left maxillary gingiva had been subjected to frozen study. The results indicated that metastasis of the previously treated RCC could not be ruled out. Subtotal maxillectomy was performed by an intraoral approach (Fig. 3). Intraoperative blood loss was only 26 ml. After surgery, there was no problems with oral intake of the patient. Although no tumor recurrence was observed in the oral cavity, our patient died 17 months after surgery because of widespread pulmonary metastases.

**Fig. 4:**
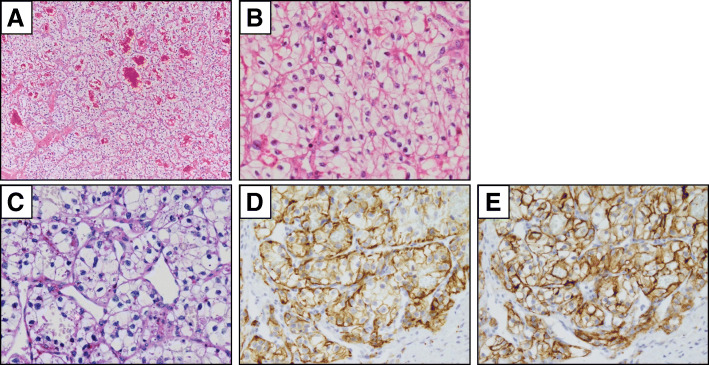
Histological findings of renal cell cancer metastasis to maxillary bone. Hematoxylin and eosin staining identified clear cell differentiation with delicate vascular stroma (**a**, ×100) and cellular atypia (**b**, ×400). Periodic acid–Schiff staining revealed positive granules in tumor cells (**c**, ×400). Immunohistochemical staining revealed positivity for AE1/3 (**d**, ×400) and CD10 (**e**, ×400)

Histologically, the neoplastic cells were arranged in an alveolar pattern with intervening delicate vascular stroma (Fig. 4a). The tumor cells displayed round to polygonal nuclei, with mild to moderate atypia, and large amounts of clear cytoplasm (Fig. 4b). Periodic acid–Schiff-positive granules were found in some tumor cells (Fig. 4c). Immunohistochemistry staining revealed that the tumor cells were positive for CD10 and AE1/3 (Fig. 4d, e). The tumor was diagnosed on histopathology as a metastasis of clear cell RCC to the maxillary bone.

## Discussion

Nearly one in three patients with RCC develops distant metastatic disease. The most common site of distant metastasis is lung (75%), followed by bone (20%), and liver (18%) [4]. Metastasis of RCC to the head and neck region is relatively rare, with a reported incidence of 15% [5]. A literature review revealed 153 patients with renal carcinoma metastasizing to the oral cavity from 1970 to 2020 [3, 5–40] (Table 1). Male patients were more frequently affected than female patients (male to female ratio, 3:1), with ages ranging between 1 and 89 years (mean age, 61.4 years). The tongue was involved in renal metastases in most patients (62 patients, 39.5%). Ninety-three patients were diagnosed with oral metastatic lesions after primary renal cancer. The duration from the onset of renal cancer to oral metastasis ranged from 2 weeks to 26 years, with an average of 4.8 years.

**Table 1:** Characteristics of patients (*N* = 153)

Gender	
Men	113
Women	38
Not described	2
Age (yr), range (mean)	1–89 (61.4)
Oral metastatic site (%)	
Tongue	62 (39.5)
Mandibular bone	30 (19.1)
Gingiva	29 (18.5)
Palate	11 (7.0)
Maxillary bone	7 (4.5)
Buccal mucosa	6 (3.8)
Lip	5 (3.2)
Floor of mouth	3 (1.9)
Metastatic site other than oral cavity (%)	
Lung	86 (59.3)
Bone	36 (24.8)
Brain	15 (10.3)
Lymph node	15 (10.3)
Liver	11 (7.6)
Adrenal gland	9 (6.2)
Skin	5 (3.4)
Mediastinum	4 (2.8)
Muscle	4 (2.8)
None	34 (23.4)
Timing of diagnosis of oral metastasis	
Before the diagnosis of primary renal carcinoma	58
After the diagnosis of primary renal carcinoma	93
Not described	2
Time^a^, range (mean)	2 w – 26 yr (4.8 yr)
Symptom	
Bleeding	27
Dysphagia	18
Pulsation	7

*w* week, *yr* year

^a^The time from the onset of primary renal carcinoma to the metastasis of oral cavity

More than three-quarters of patients with oral metastasis from renal carcinoma also exhibited other metastatic lesions, primarily lung metastases (86 patients, 59.3%) (Table 1). Because of the high rate of lung metastases, the prognosis is reported to be very poor; most patients die within the first year after diagnosis [3]. Regarding treatments, surgery is often recommended due to the ability of metastatic RCC to be resistant to radiotherapy and pharmacotherapy, including chemotherapy, molecularly targeted therapy, or immunotherapy [41]. However, considering the poor prognosis of oral metastasis of renal cancer, clinicians have to weigh the benefits of surgery and the disadvantages of not having it.

Oral metastatic lesions of renal cancer are known to undergo extremely rapid enlargement, which results in severe symptoms and a decline in quality of life. Dysphagia was observed in 18 patients (Table 1). Obstruction of the upper airways due to rapid growth of oral metastasis led to tracheotomy in one patient [18] and death in one patient [16]. Mazeron *et al.* described a patient with RCC metastasis to the tongue, who initially decided against surgery due to the poor prognosis. However, doctors were later forced to perform surgery because of a rapid increase in the size of the intraoral mass and resistance of the tumor to chemoradiotherapy [23]. In the present case, our patient began to feel pain because of the rapid increase of the intraoral mass. It was possible that dysphagia or obstruction of upper airway may have occurred without surgery, suggesting that surgical therapy can maintain quality of life of the patient.

Among 153 patients with oral metastasis of RCC described in the literature, the treatment and outcome of the oral lesion were described for 78 patients, which are summarized in Table 2. Surgery was performed in most patients (53 cases), and the local control rate was greater than 90%. In contrast, the local control rates of radiotherapy, pharmacotherapy, and palliative surgery (debulking and cryosurgery) ranged from 33.3% to 66.7%. Considering the high ratio of local control after surgery, surgical therapy before further disease progression may be the first choice for patients with oral metastasis of RCC.

**Table 2:** Therapeutic outcomes of oral metastatic lesion of renal cell cancer. (*N* = 78)

Treatment	Outcome		
	Controlled^**a**^	Uncontrolled	Control ratio (%)
S	41	2	95.2
S + P/R	12	1	92.3
PS	3	3	50.0
R	4	3	57.1
P	2	4	33.3
P + R	2	1	66.7

*P* pharmacotherapy, *P/R* pharmacotherapy and/or radiotherapy, *PS* palliative surgery, *R* radiotherapy, *S* surgery

^a^“Controlled” indicates no local recurrence after surgery, or regression of tumor after PS/P/R

However, hemorrhage was observed in many patients because RCC is characterized by rich blood vessels, which makes surgery difficult. Pulsation was observed in seven patients at the first visit [7, 9–11, 16]. Three of these patients exhibited pulsation in the maxillary bone [11] (Tables 1 and 3). In our patient, arteriovenous malformation was clinically suspected due to pulsation. Past reports indicated that two patients with mandibular lesions were also clinically diagnosed with vascular malformation, then diagnosed with RCC metastases upon pathological examination [8, 10]. Twenty-seven patients exhibited bleeding at the first visit or during follow-up, which resulted in death in one case [31]. Ten patients had difficulty with hemostasis during biopsy or surgery [7, 12, 13, 16, 25, 27, 34, 36, 39]. Three patients underwent preoperative vascular embolization or ligation, which did not lead to complications during surgery [9, 10, 13]. Our experience with the present patient also indicated that preoperative vascular embolization is helpful in preventing massive hemorrhage during biopsy and surgery for pulsatile oral metastasis of RCC. We believe that surgical therapy after vascular embolization can improve or maintain quality of life for the patients with oral metastasis of renal carcinoma, even if they are late stage oncological patients.

**Table 3:** Summary of the reported cases of renal cell cancer metastasis in the maxillary bone

Case	Age	Gender	Pulsation	Treatment	Outcome		
					Oral metastasis	Prognosis^**a**^	Ref.
1	66	F	–	S	ND	ND	9
2	58	M	+	E	Uncontrolled	Dead, 2 m	20
3	73	M	+	E	NS	Dead, 1.5 m	20
4	53	F	–	R + E + S	Controlled	Alive, 14 m	26
5	60	M	–	S	ND	ND	52
6	54	M	–	CR	ND	Dead, 11 m	59
7	89	M	+	E + S	Controlled	Dead, 19 m	^b^

*CR* chemoradiotherapy, *E* embolization, *F* female, *M* male, *ND* not described, *NS* no symptom with disease, *R* radiotherapy, *S* surgery

^a^ Numbers are length of follow-up in months

^b^ Present case

## Conclusions

Although oral metastasis of RCC has a poor prognosis due to the presence of concurrent disseminated metastases, surgical therapy may be recommended because of its high local control rate and ability to maintain quality of life. Preoperative vascular embolization is considered to be effective to reduce intraoperative hemorrhage, which leads to safe operation.

## Data Availability

All data generated or analysed during this study are included in this published article.

## Abbreviation

RCC: Renal cell carcinoma
